# Advances in prosthetic technology: a perspective on ethical considerations for development and clinical translation

**DOI:** 10.3389/fresc.2023.1335966

**Published:** 2024-01-16

**Authors:** Hayden Gavette, Cody L. McDonald, Kristin Kostick-Quenet, Ashley Mullen, Bijan Najafi, M. G. Finco

**Affiliations:** ^1^ Orthotics and Prosthetics Program, School of Health Professions, Baylor College of Medicine, Houston, TX, United States; ^2^ Department of Rehabilitation Medicine, University of Washington, Seattle, WA, United States; ^3^ Center for Medical Ethics and Health Policy, Baylor College of Medicine, Houston, TX, United States; ^4^ Interdisciplinary Consortium on Advanced Motion Performance Lab (iCAMP), Department of Surgery, Baylor College of Medicine, Houston, TX, United States

**Keywords:** ethics, perspective, prosthesis, amputees, rehabilitation, clinic, research, translation

## Abstract

Technological advancements of prostheses in recent years, such as haptic feedback, active power, and machine learning for prosthetic control, have opened new doors for improved functioning, satisfaction, and overall quality of life. However, little attention has been paid to ethical considerations surrounding the development and translation of prosthetic technologies into clinical practice. This article, based on current literature, presents perspectives surrounding ethical considerations from the authors' multidisciplinary views as prosthetists (HG, AM, CLM, MGF), as well as combined research experience working directly with people using prostheses (AM, CLM, MGF), wearable technologies for rehabilitation (MGF, BN), machine learning and artificial intelligence (BN, KKQ), and ethics of advanced technologies (KKQ). The target audience for this article includes developers, manufacturers, and researchers of prosthetic devices and related technology. We present several ethical considerations for current advances in prosthetic technology, as well as topics for future research, that may inform product and policy decisions and positively influence the lives of those who can benefit from advances in prosthetic technology.

## 1 Introduction

As prosthesis use has increased, technology has continued to advance, resulting in many scientific breakthroughs over the last decade. A few examples include haptic feedback to restore sensation (1, 2), componentry that can provide active power (3) and machine learning for prosthetic control (1, 3). These and similar advances in prosthetic technology have the potential to revolutionize prosthetic care (2, 3); however, ethical concerns of development and implementation into current clinical practice are often not discussed, contributing to a widening gap between research and clinical practice as well as wasted research costs. For example, actively powered knee and ankle prostheses have encountered multiple challenges (e.g., being too heavy, being too bulky, being inefficient, not providing enough power to actively support the patient's weight and activity) that have limited commercialization (4). Providing developers and manufacturers of prosthetic technology an overview of ethical concerns related to development and clinical translation could help prevent wasted research costs and maximize potential benefits of prosthetic technology as advances continue.

In this perspective article, we present the author's viewpoints on several ethical considerations for advances in prosthetic technology, as well as topics for future research. Specifically, we discuss topics within device development and translation to clinical practice. While not an exhaustive list, we hope the ethical considerations discussed in these sections can help bridge existing gaps between clinicians and developers, manufacturers, and researchers, to ultimately inform user-centered design, establish policy guidelines, and reduce wasted research costs. Most importantly, proactively addressing ethical considerations from both a research and clinical perspective can help ensure that people who receive prosthetic care actually benefit from current and continued advancements in prosthetic technology.

## 2 Device development

The development of advanced prosthetic technology is generally conducted with little input from users or clinicians. In this section, we highlight the importance of considering user-centered design principles, participatory action research, reported needs of prosthesis users, and clinician perspectives to optimize device development.

### 2.1 Utilize user-centered design and participatory action research

User-centered design, also termed co-creation or human-centered design and along the same paradigms as value sensitive design (5), is the process in which developers include the needs, values, opinions, and concerns of end-users throughout the design and implementation of a novel idea or product (6). Without user-centered design in prosthetics, developers risk wasting resources towards the production of new devices that may be unusable or undesirable among end-users. More importantly, participatory action research allows a shift from thinking of people as “end-users” towards integrating them as equal members of the team developing the technology (i.e., making technology *with* people instead of *for* them), ensuring development from idea generation to implementation is relevant to their lived experiences (7). Several studies have reported the perspectives of prosthesis users in the context of current clinically available prosthetic technology, such as cosmesis (making the prosthesis aesthetically pleasing), prosthetic fit/comfort, functionality, and specific prosthetic componentry that may help clinicians provide the best services for their patients (8–21). However, there is limited research focusing on user perspectives regarding future technology to guide the development of new prosthetic devices (22–25). As advanced technologies continue to enter the field of prosthetics, these user perspectives must be reported to ensure the technologies are beneficial before they are made readily available. Yet, user expectations for prosthetic technology may be unrealistically high and unattainable, so it is important to properly educate individuals on inherent trade-offs of design to collect informed perspectives. Of equal concern, including a diverse user group that is representative of the larger target audience is challenging, yet must be considered when collecting these perspectives. Additionally, user-centered design frameworks (6, 23, 26–28), the Usability Metric for User Experience (UMUX) (29), and the Technology Acceptance Model (TAM) (30) should be utilized in future studies to guide new technology design and assess its acceptance by users.

### 2.2 Determine user needs

In a recent review of lower limb prosthesis (LLP) user needs, pain reduction, mobility, social integration, independence, and the ability to walk were the *most frequently reported* needs while safety was reported as another important need (31). Some of the advanced technology currently used in the clinical setting has resulted in improvements in these areas (32–35) but further development is necessary to meet these needs (31). Although limited, user-centered design as a component of advancing LLP technology has been reported (22, 23), Fanciullacci et al. found that transfemoral amputees reported they would like their powered robotic prosthesis to assist in ascending stairs and inclines, but not running (22). Similarly, Beckerle et al. utilized a human-machine-centered design process that considers both the technical and user needs, weighs their importance in the overall design of the technology, and proposes the priorities to guide the development process (23). Approaches similar to these studies should be implemented in the development of advanced LLP technology to help ensure the technology's acceptance and success in the hands of the users. Further, future LLP technology must meet or exceed the benefits of current technologies in the realm of end-user mobility, independence, comfort, and safety to promote adoption.

Upper limb prosthesis (ULP) users have reported unique needs compared to lower-limb prosthesis users. A recent review reported ULP users require more functionality (e.g., grasping, manipulation, and strength), better cosmesis, and better comfort out of their devices regardless of device type (body powered, myoelectric, passive) or level of limb loss/difference (36). Additionally, users request sensory feedback, higher battery and electrode reliability and durability, less dependence on visual attention while using their prosthesis, accurate anthropomorphic dimensions, less heat retention, and less motor noise (36). Although more recent developments in ULP technology have sought to resolve these issues, these needs are nearly identical to those reported in a study published over 20 years ago (37). Similarly, device abandonment in ULP users has been a concern for decades, yet current prosthetic technology still has not improved abandonment rates (38, 39). In a recent survey, 44% of ULP users rejected the use of their prosthesis despite almost 93% of them having been prescribed one of the most advanced ULP technologies clinically available (myoelectric control) (38). Reasons for abandonment are due to discomfort (too heavy, too hot, causing excessive sweating), non-ideal function (inhibited control, no sensory feedback), and users being more independent without a prosthesis (39). In addition, advanced prosthetic technology requires various levels of training to deliver optimal user benefits, and overlooking prosthetic training can lead to abandonment (40). Developers and manufacturers can combat abandonment and excessive training needs by developing more intuitive control mechanisms, rather than entirely new devices, and offer clear instructions that physical and occupational therapists can also use to help patients adapt to their devices. As much as ULP technology development has grown over recent decades, it is clear that more must be done to meet the needs of prosthesis users. Further, assessment of the needs and perspectives of prosthetic users regarding advanced ULP technology is sparse. Engdahl et al. found that users are more interested in current clinically available, non-invasive myoelectric prostheses and the ability to complete more basic functions, rather than undergo surgery or the ability to complete advanced functions that would be included with new technologies (24). However, Kelley et al. suggests that users are willing to accept the risks associated with the new technologies if there is a significant functional benefit such as sensory feedback, improved user control, and reduced training time and maintenance (25). Nonetheless, these findings conclude that user perspectives towards advanced ULP technologies must be researched further to help guide technology development.

### 2.3 Include clinician perspectives

Prosthetic device development should also involve the perspectives of the clinicians, (e.g., prosthetists, physical therapists, occupational therapists, and others) who are members of the interprofessional healthcare team. In a focus group involving clinicians, users, researchers, and device manufacturers, Klute et al. determined fit, comfort, function, performance, and stability were important LLP user needs that the authors suggest can be improved by developing standardized outcome measures (41). Additionally, non-technical features, like improved patient education about the rehabilitation process, improved communication, improved evidence-based guidelines, and improved patient support systems, are just as important (41). Rekant et al. investigated clinician perspectives on current and prospective ULP technology and found that clinicians emphasized the user's needs for completing activities of daily living, participating in hobbies, device reliability and safety, in-hand object manipulation, finger flexion/extension, greater wrist range of motion, and thumb abduction/adduction (42). However, compared to users, clinicians were more skeptical of invasive surgeries (42). Additionally, since prosthetists in the US are reimbursed per device rather than per clinical service, prosthetists often must consider a business perspective that may conflict with their clinical perspective. Nonetheless, further information regarding clinician perspectives on and needs for advanced prosthetic technology is necessary to guide technology development.

### 2.4 Promote health equity

Promoting health equity can help ensure people who use prostheses have access to advanced technologies that can improve their quality of life (43). To promote health equity in prosthetic design, it's critical to acknowledge that socioeconomic factors (e.g., age, race, ethnicity, gender, living in a rural environment) and other social determinants of health (e.g., racism) can contribute to health disparities in amputation rates, as well as prosthetic technology development and access (38, 43–53). Additionally, especially in the US, reduced access to quality health insurance and a lack of affordability of copays and deductibles have been found to widen disparities (54–56). It is possible that advanced prosthetic technology could continue to widen these existing health disparities. For instance, technology often requires a stable internet connection, compatible hardware and software, as well as technology literacy, training, and technical support for effective use. However, current billing practices in prosthetics dictate that any follow-up care is bundled into a lump-sum payment for the device, and providers are not reimbursed for follow-up care outside of this base rate. Additionally, people who are older adults, belong to systematically marginalized groups, or live in rural communities are often underrepresented in prosthetics literature (57–59), including technology development. To elucidate health disparities, researchers must first collect and report detailed socioeconomic information, as a recent review determined 84% of the 420 manuscripts reviewed did not report race or ethnicity of the participants (60). Collecting and reporting detailed socioeconomic information is an essential first step to begin understanding and addressing existing disparities. Researchers can also pursue topics that center people who are underrepresented in current prosthetics literature, and use recruitment strategies (i.e., participant payment for travel and/or childcare) to mitigate participation barriers and help ensure development and subsequent access to technologies are equitable.

## 3 Translation to clinical practice

Clinicians and researchers must collaborate to integrate new advanced prosthetic technology into the market and clinical practice, ensuring the greatest benefit to the user, justifying the resources spent to develop the technology, and advancing the field as a whole (61–65). Making novel prosthetic technology readily available in clinical practice requires a sustained effort of numerous resources over multiple years. For instance, microprocessor knees (MPKs) began development in the 1980's (66–68), were not commercially available in the US until 1999, and were not covered by Medicare until 2005 (69). Additionally, many patient and situational factors affect prosthetic prescription in clinical practice. For example, MPKs may not be suitable for some patients, such as individuals who are not cognitively capable of using and taking care of the MPK (70). Further, some patients, such as those classified as limited community ambulators (K2 Medicare functional classification level), may be unable to receive an MPK due to insurance coverage restrictions, though current research has demonstrated benefits to this population (68, 70). This section discusses practical considerations and potential barriers of translating new advanced prosthetic technology into clinical practice.

### 3.1 Understand reimbursement and coverage

Arguably the most critical aspect of technology translation into prosthetic patient care is device coverage. To prosthetic users and clinicians alike, cost is a crucial concern and must be accounted for in prosthetic technology development (31, 36, 41, 42, 71). As all authors are based in the US, only US coverage guidelines will be discussed, though international challenges in prosthetic coverage have also been reported (72). Kannenberg et al. discuss how insurance companies (including the Centers for Medicare and Medicaid services) have recently called for greater evidence with high-quality methods to document the clinical benefit of prosthetic technology and guide payment rates (72, 73). Although it is difficult to mask participants and randomize study groups in prosthetics research, high-quality evidence is crucial to justify the need and subsequent reimbursement of prosthetic technology. As manufacturers and developers continue to produce novel technologies, the cost of high-quality research can be priced into the product to account for this need. Additionally, insurance companies can dictate whether cheaper technologies provide equal benefit and will therefore be sufficient for the patient (72). Thus, new prosthetic technology must have a documented added benefit in order to receive adequate reimbursement, and developers and manufacturers should await the publication of this documented benefit prior to marketing the technology. Negotiating reimbursement with third party payers would also be easier if developers and manufacturers defined a specific target population rather than using the traditional yet vague K-Level classifiers or “product for all” approach. Furthermore, the ability of prosthetists to bill for time spent manufacturing, aligning, repairing, or otherwise managing a prosthetic device is limited. Thus, it may be unwise to develop prosthetic technology that requires extensive maintenance as clinicians may reject it on the basis of losing time, effort, and money. Cosmetic devices, though they have been documented to positively impact personal identity and overall quality of life (8, 17, 31, 74–78) are generally regarded as not medically necessary and are not covered by insurance. Despite the struggle to obtain suitable reimbursement for prosthetic devices, clinicians and patients rely on insurance coverage as any remaining costs must be covered out-of-pocket by the patient or sometimes charitable organizations. Finally, it is important to ensure that new prosthetic technology is accessible to all individuals regardless of financial status. Though they may not provide all the same benefits as their higher-end counterparts, more cost-effective options are necessary to meet the needs of all individuals. Ultimately, it is important to keep these funding structures in mind during the development of new prosthetic technology, as device payment is needed for device utilization. If not already doing so, developers could also help advocate for changes in the billing and coding system to improve coverage and reimbursement.

### 3.2 Abide by regulatory and education standards

An additional step in transitioning new prosthetic technology to the market is abiding by regulatory, manufacturing, and education standards. For instance, the use of digital technology to fabricate prostheses has risen with the advent of computer aided design and manufacturing and 3D printing technology. Both people receiving prosthetic care and prosthetists have expressed concerns regarding durability, safety, and aesthetics of 3D printed lower-limb prosthetic sockets (79). Compliance with manufacturing standards, such as ISO/TC 168 (80) and FDA 21CFR890 (81), ensure the device is safe and suitable for use. Additionally, while digital technologies can be excellent tools to integrate into clinical practice, concern remains over a lack of certification regulations for people who attempt to fit prosthetic devices who have not received the education (currently a Master's degree) or who are not subject to regulations (state licensure or certification required of prosthetists). Though a global shortage in training programs and certified prosthetists is evident (82–84), governing bodies such as the World Health Organization (85), the International Society for Prosthetics and Orthotics (86), and the National Commission on Orthotic and Prosthetic Education (87) have advocated for increased education standards and improved prosthetist training. Furthermore, the emphasis of evidence-based practice in prosthetic education (84, 88), equipping prosthetist educators with tools for effective teaching (84, 89), and utilizing internships and residencies to transition students into skilled clinicians (84, 90) have also improved prosthetic education. Companies, individuals, and researchers who are not prosthetists can seek to include certified prosthetists in their business model or research team to ensure the safety of prosthesis users. Following ethical design, regulatory, and manufacturing processes not only provides protection of the technology and its developers from liability issues, but also improves user safety and user trust in the technology, further improving its acceptance and adoption in clinical practice.

### 3.3 Encourage patient autonomy

Patient autonomy and informed consent are of utmost importance in clinical practice and should, likewise, be of importance to researchers in prosthetic technology development. Complete transparency about the design process, intended functionality, benefits and drawbacks, costs, and maintenance requirements for prosthetic devices should always be conveyed by researchers, to help clinicians convey these aspects transparently to patients. Additionally, regardless of whether a patient will be able to afford or effectively utilize a specific prosthetic technology, the patient is still entitled to know all of their options. Prosthetists may act as gatekeepers to device options, potentially only presenting prosthetic technology that they deem appropriate. Decisions about appropriate prosthetic technology may be influenced by implicit and explicit biases. To bridge this gap, shared decision-making models help clinicians improve communication, understand patient values, utilize their clinical experience, and clarify the prosthetic journey for the patient (91–94). Researchers and developers can develop more decision-making models as well as provide greater information and education on new technologies to aid clinicians in this endeavor. Novel technologies are commonly more complex than previous technologies, so developers must find a means of helping clinicians fully explain these complexities to patients. Decision-making aids are one tool within knowledge translation, which is the field of study dedicated to expediting the implementation of research into clinical practice (95). Despite the value that knowledge translation research could bring to effective clinical translation (96), it remains underexamined by researchers in prosthetics literature. Lastly, for future implications of advanced prosthetic technology, it is important to inform patients that their data (collected for monitoring and secondary data use) may be used in ways that are not known at the time they are giving consent.

### 3.4 Consider data collection and privacy

While not yet integrated into standard clinical practice, several studies have demonstrated that wearable sensors, machine learning, and artificial intelligence could potentially be used in clinical practice to improve prosthetic care (97–100). However, many challenges still exist in integrating these technologies into clinical practice (e.g., privacy concerns with data collection and storage, maintaining software updates, data collection and storage, cost-effectiveness, clinician scope of practice, health equity) (101–105). While standards and guidelines are still emerging, commitment and regulation from developers is crucial, yet difficult to enforce. Researchers and policymakers in prosthetics can look at practical applications, such as governance models, that other fields have recently raised (101). While some advances that could utilize machine learning or artificial intelligence (e.g., brain-computer interfaces, ability to feel temperature or pressure) have not yet left a research setting, they have clear applications for improving functionality (e.g., increased perceptions of prosthetic embodiment, more intuitive control of the device) or remotely monitoring rehabilitation. It is important to consider how integrating these technologies into prosthetic devices could inform clinical decision-making to further prevent complications, manage comorbidities, and improve long-term health of prosthesis users. Specifically, future studies could determine how advanced prosthetic technologies could help monitor and predict rehabilitation adverse events (e.g., falls) to improve overall patient care, while also considering how this could influence clinician scope of practice. Additionally, since limb loss and difference are expected to be permanent disabilities, devising methods to make long-term digital healthcare accessible are also crucial for patient success, and promoting health equity (106, 107).

## 4 Summary

**Figure 1:**
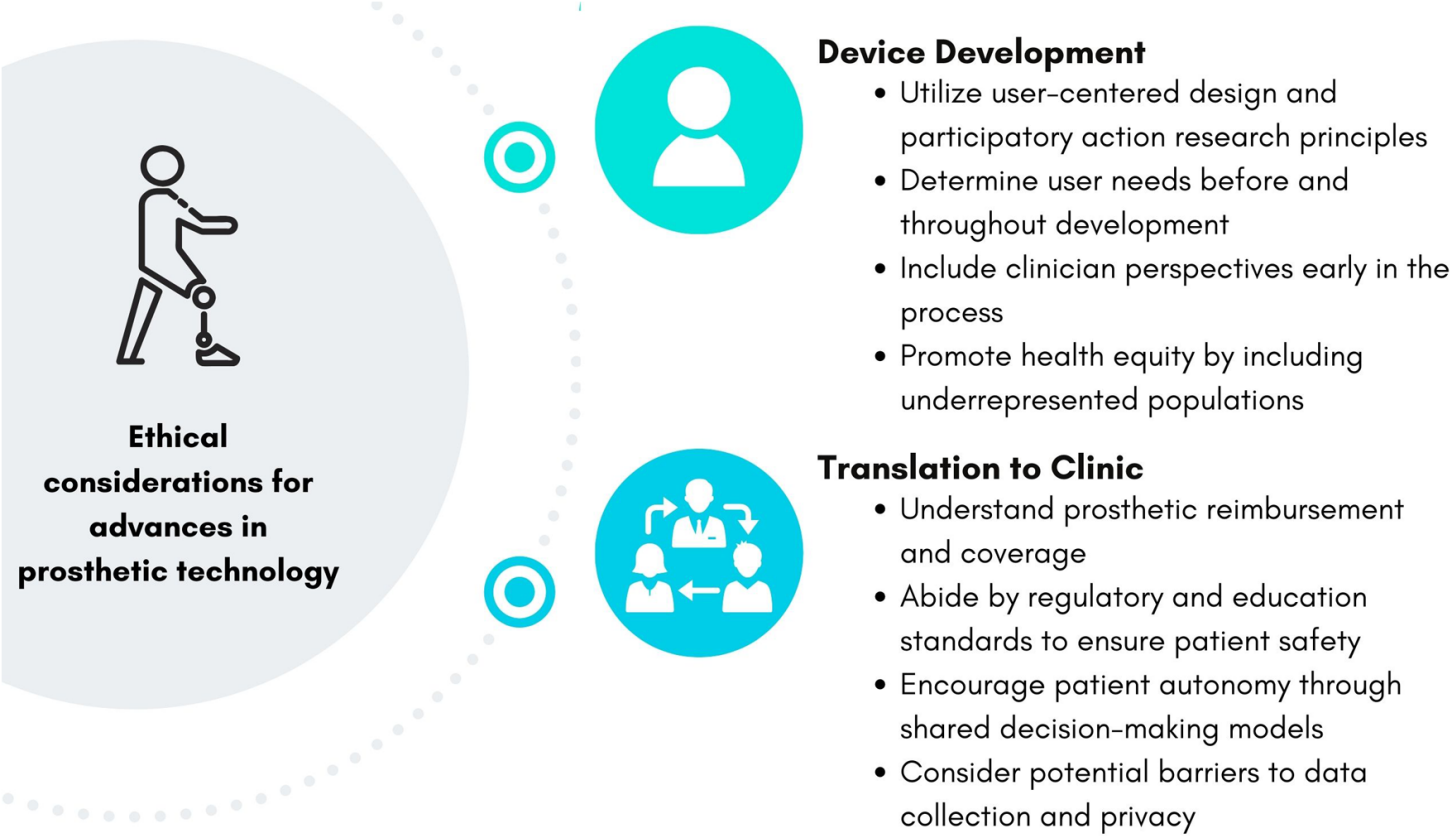
Ethical considerations and action items for the development and translation of prosthetic technology.

Figure 1 summarizes the ethical considerations and action items discussed in this perspective article. Developers, manufacturers, and researchers can implement these considerations throughout the process of developing advanced prosthetic technology. Utilizing user-centered design frameworks, as well as centering the needs of people with limb loss and difference and clinician perspectives, are crucial to ensure prosthetic technology will be beneficial to those who will use it. Additionally, determining the need for and benefit of a new prosthetic technology can ultimately prevent wasted research costs. Understanding the barriers to translating advances in prosthetic technology to clinical care before and throughout development can help ensure the technology will be safe to use, accessible, and successful on the market to improve patient outcomes.

## 5 Conclusion

Although each of the points summarized in Figure 1 are crucial to consider throughout development of novel prosthetic technology, many may conflict. For instance, it may be difficult to balance the need for technology that abides by regulatory standards and employs high-quality research, but also remains inexpensive and accessible to all potential users. Further, members of interdisciplinary teams developing new prosthetic technology may have varying priorities, which may also differ by situation or change over time. Research is needed to incorporate these various design criteria into priority-ranking frameworks, like the one proposed by Beckerle et al. (23), to help developers, manufacturers, and researchers realistically implement these considerations as prosthetic technology advances. Additionally, decision-making and decisional support guides must be developed to aid clinicians in understanding and incorporating new technologies into their practices. Advances in prosthetic technology have the potential to revolutionize care for prosthetic patients, but it is imperative that these technologies are designed ethically and in consideration of end users.

## 6 Author positionality

Most authors of this article are prosthetists and/or researchers of people with limb loss and difference, as detailed in the abstract. It is essential to note that none of the authors have limb loss or difference, so we have not personally encountered consequences of ethical barriers related to prosthetic technology. This article represents an effort to critically examine and evaluate ethical barriers related to prosthetic technology in our professional community. We aim to foster greater transparency, equity, and inclusivity throughout the development and translation of prosthetic technology in our community and in our own work.

## Data Availability

The original contributions presented in the study are included in the article/Supplementary Material, further inquiries can be directed to the corresponding author.

## References

[1] Dupont PE Nelson BJ Goldfarb M Hannaford B Menciassi A O’Malley MK A decade retrospective of medical robotics research from 2010 to 2020. Sci Robot. (2021) 6(60):eabi8017. 10.1126/scirobotics.abi8017 34757801 PMC8890492

[2] Trent L Intintoli M Prigge P Bollinger C Walters LS Conyers D A narrative review: current upper limb prosthetic options and design. Disabil Rehabil Assist Technol. (2020) 15(6):604–13. 10.1080/17483107.2019.1594403 30973275

[3] Asif M Tiwana M Khan U Qureshi W Iqbal J Rashid N Advancements, trends and future prospects of lower limb prosthesis. IEEE Access. (2021) 9:1. 10.1109/ACCESS.2021.3086807

[4] Windrich M Grimmer M Christ O Rinderknecht S Beckerle P. Active lower limb prosthetics: a systematic review of design issues and solutions. BioMed Eng OnLine. (2016) 15(3):140. 10.1186/s12938-016-0284-9 28105948 PMC5249019

[5] Friedman B Kahn PH Borning A Huldtgren A. Value sensitive design and information systems. In: Doorn N Schuurbiers D van de Poel I Gorman ME, editors. Early Engagement and New Technologies: Opening Up the Laboratory. Dordrecht: Springer Netherlands (2013). p. 55–95. 10.1007/978-94-007-7844-3_4

[6] Schumacher R Lowry S. *(NISTIR 7741) NIST Guide to the Processes Approach for Improving the Usability of Electronic Health Records*. (2010). Available at: https://tsapps.nist.gov/publication/get_pdf.cfm?pub_id=907313 (Accessed November 09, 2023).

[7] Baum F MacDougall C Smith D. Participatory action research. J Epidemiol Community Health. (2006) 60(10):854–7. 10.1136/jech.2004.028662 16973531 PMC2566051

[8] Cairns N Murray K Corney J McFadyen A. Satisfaction with cosmesis and priorities for cosmesis design reported by lower limb amputees in the United Kingdom: instrument development and results. Prosthet Orthot Int. (2014) 38(6):467–73. 10.1177/0309364613512149 24327666 PMC4230545

[9] Bosman CE van der Sluis CK Geertzen JHB Kerver N Vrieling AH. User-relevant factors influencing the prosthesis use of persons with a transfemoral amputation or knee-disarticulation: a meta-synthesis of qualitative literature and focus group results. PLoS One. (2023) 18(1):e0276874. 10.1371/journal.pone.0276874 36649233 PMC9844830

[10] Pezzin LE Dillingham TR Mackenzie EJ Ephraim P Rossbach P. Use and satisfaction with prosthetic limb devices and related services. Arch Phys Med Rehabil. (2004) 85(5):723–9. 10.1016/j.apmr.2003.06.002 15129395

[11] Dillingham TR Pezzin LE MacKenzie EJ Burgess AR. Use and satisfaction with prosthetic devices among persons with trauma-related amputations: a long-term outcome study. Am J Phys Med Rehabil. (2001) 80(8):563–71. 10.1097/00002060-200108000-00003 11475475

[12] Baars EC Schrier E Dijkstra PU Geertzen JHB. Prosthesis satisfaction in lower limb amputees: a systematic review of associated factors and questionnaires. Medicine (Baltimore). (2018) 97(39):e12296. 10.1097/MD.0000000000012296 30278503 PMC6181602

[13] Luza LP Ferreira EG Minsky RC Pires GKW da Silva R. Psychosocial and physical adjustments and prosthesis satisfaction in amputees: a systematic review of observational studies. Disabil Rehabil Assist Technol. (2020) 15(5):582–9. 10.1080/17483107.2019.1602853 31012753

[14] Richardson A Dillon M. User experience of transtibial prosthetic liners: a systematic review. Prosthet Orthot Int. (2016) 41:6–18. 10.1177/0309364616631343 26932981

[15] Jang CH Yang HS Yang HE Lee SY Kwon JW Yun BD A survey on activities of daily living and occupations of upper extremity amputees. Ann Rehabil Med. (2011) 35(6):907–21. 10.5535/arm.2011.35.6.907 22506221 PMC3309384

[16] Biddiss EA Chau TT. Upper limb prosthesis use and abandonment: a survey of the last 25 years. Prosthet Orthot Int. (2007) 31(3):236–57. 10.1080/03093640600994581 17979010

[17] Ritchie S Wiggins S Sanford A. Perceptions of cosmesis and function in adults with upper limb prostheses: a systematic literature review. Prosthet Orthot Int. (2011) 35(4):332–41. 10.1177/0309364611420326 21960052

[18] Walker MJ Goddard E Stephens-Fripp B Alici G. Towards including end-users in the design of prosthetic hands: ethical analysis of a survey of Australians with upper-limb difference. Sci Eng Ethics. (2020) 26(2):981–1007. 10.1007/s11948-019-00168-2 31832867

[19] Ju N Lee KH Kim MO Choi Y. A user-driven approach to prosthetic upper limb development in Korea. Healthcare (Basel). (2021) 9(7):839. 10.3390/healthcare9070839 34356217 PMC8303819

[20] Jones H Dupan S Dyson M Krasoulis A Kenney LPJ Donovan-Hall M Co-creation and user perspectives for upper limb prosthetics. Front Neurorobot. (2021) 15:689717. 10.3389/fnbot.2021.689717 34305564 PMC8299561

[21] Kerver N van der Sluis CK van Twillert S Krabbe PFM. Towards assessing the preferred usage features of upper limb prostheses: most important items regarding prosthesis use in people with major unilateral upper limb absence—a Dutch national survey. Disabil Rehabil. (2022) 44(24):7554–65. 10.1080/09638288.2021.1988734 34813394

[22] Fanciullacci C McKinney Z Monaco V Milandri G Davalli A Sacchetti R Survey of transfemoral amputee experience and priorities for the user-centered design of powered robotic transfemoral prostheses. J Neuroeng Rehabil. (2021) 18(1):168. 10.1186/s12984-021-00944-x 34863213 PMC8643009

[23] Beckerle P Christ O Schürmann T Vogt J Von Stryk O Rinderknecht S. A human-machine-centered design method for (powered) lower limb prosthetics. Rob Auton Syst. (2017) 95:1–12. 10.1016/j.robot.2017.05.004

[24] Engdahl SM Christie BP Kelly B Davis A Chestek CA Gates DH. Surveying the interest of individuals with upper limb loss in novel prosthetic control techniques. J Neuroeng Rehabil. (2015) 12(1):53. 10.1186/s12984-015-0044-2 26071402 PMC4465617

[25] Kelley MA Benz H Engdahl S Bridges JFP. Identifying the benefits and risks of emerging integration methods for upper limb prosthetic devices in the United States: an environmental scan. Expert Rev Med Devices. (2019) 16(7):631–41. 10.1080/17434440.2019.1626231 31145868

[26] Rodriguez NM Burleson G Linnes JC Sienko KH. Thinking beyond the device: an overview of human- and equity-centered approaches for health technology design. Annu Rev Biomed Eng. (2023) 25:257–80. 10.1146/annurev-bioeng-081922-024834 37068765 PMC10640794

[27] Göttgens I Oertelt-Prigione S. The application of human-centered design approaches in health research and innovation: a narrative review of current practices. JMIR Mhealth Uhealth. (2021) 9(12):e28102. 10.2196/28102 34874893 PMC8691403

[28] Domínguez-Ruiz A López-Caudana EO Lugo-González E Espinosa-García FJ Ambrocio-Delgado R García UD Low limb prostheses and complex human prosthetic interaction: a systematic literature review. Front Robot AI. (2023) 10:1032748. 10.3389/frobt.2023.1032748 36860557 PMC9968924

[29] Borsci S Buckle P Walne S. Is the LITE version of the usability metric for user experience (UMUX-LITE) a reliable tool to support rapid assessment of new healthcare technology? Appl Ergon. (2020) 84:103007. 10.1016/j.apergo.2019.103007 31785449

[30] Rahimi B Nadri H Lotfnezhad Afshar H Timpka T. A systematic review of the technology acceptance model in health informatics. Appl Clin Inform. (2018) 9(3):604–34. 10.1055/s-0038-1668091 30112741 PMC6094026

[31] Manz S Valette R Damonte F Avanci Gaudio L Gonzalez-Vargas J Sartori M A review of user needs to drive the development of lower limb prostheses. J Neuroeng Rehabil. (2022) 19(1):119. 10.1186/s12984-022-01097-1 36335345 PMC9636812

[32] Hebert JS Rehani M Stiegelmar R. Osseointegration for lower-limb amputation: a systematic review of clinical outcomes. JBJS Rev. (2017) 5(10):e10. 10.2106/JBJS.RVW.17.00037 29087966

[33] Kunutsor SK Gillatt D Blom AW. Systematic review of the safety and efficacy of osseointegration prosthesis after limb amputation. Br J Surg. (2018) 105(13):1731–41. 10.1002/bjs.11005 30307036

[34] Hahn A Bueschges S Prager M Kannenberg A. The effect of microprocessor controlled exo-prosthetic knees on limited community ambulators: systematic review and meta-analysis. Disabil Rehabil. (2022) 44(24):7349–67. 10.1080/09638288.2021.1989504 34694952

[35] Thibaut A Beaudart C Maertens DE Noordhout B Geers S Kaux JF Impact of microprocessor prosthetic knee on mobility and quality of life in patients with lower limb amputation: a systematic review of the literature. Eur J Phys Rehabil Med. (2022) 3:452–61. 10.23736/S1973-9087.22.07238-0 PMC9987462 35148043

[36] Cordella F Ciancio AL Sacchetti R Davalli A Cutti AG Guglielmelli E Literature review on needs of upper limb prosthesis users. Front Neurosci. (2016) 10:209. 10.3389/fnins.2016.00209 27242413 PMC4864250

[37] Atkins DJ Heard DCY Donovan WH. Epidemiologic overview of individuals with upper-limb loss and their reported research priorities. J Prosthet Orthot. (1996) 8(1):2–11. 10.1097/00008526-199600810-00003

[38] Salminger S Stino H Pichler LH Gstoettner C Sturma A Mayer JA Current rates of prosthetic usage in upper-limb amputees—have innovations had an impact on device acceptance? Disabil Rehabil. (2022) 44(14):3708–13. 10.1080/09638288.2020.1866684 33377803

[39] Smail LC Neal C Wilkins C Packham TL. Comfort and function remain key factors in upper limb prosthetic abandonment: findings of a scoping review. Disabil Rehabil Assist Technol. (2021) 16(8):821–30. 10.1080/17483107.2020.1738567 32189537

[40] Resnik L Meucci MR Lieberman-Klinger S Fantini C Kelty DL Disla R Advanced upper limb prosthetic devices: implications for upper limb prosthetic rehabilitation. Arch Phys Med Rehabil. (2012) 93(4):710–7. 10.1016/j.apmr.2011.11.010 22464092

[41] Klute GK Kantor C Darrouzet C Wild H Wilkinson S Iveljic S Lower-limb amputee needs assessment using multistakeholder focus-group approach. J Rehabil Res Dev. (2009) 46(3):293–304. 10.1682/JRRD.2008.02.0031 19675983

[42] Rekant J Fisher LE Boninger ML Gaunt RA Collinger JL. Amputee, clinician, and regulator perspectives on current and prospective upper extremity prosthetic technologies. Assist Technol. (2023) 35(3):258–70. 10.1080/10400435.2021.2020935 34982647

[43] Pasquina CPF Carvalho AJ Sheehan TP. Ethics in rehabilitation: access to prosthetics and quality care following amputation. AMA J Ethics. (2015) 17(6):535–46. 10.1001/journalofethics.2015.17.6.stas1-1506 26075981

[44] Saeed SA Masters RM. Disparities in health care and the digital divide. Curr Psychiatry Rep. (2021) 23(9):61. 10.1007/s11920-021-01274-4 34297202 PMC8300069

[45] Turner S Belsi A McGregor AH. Issues faced by prosthetists and physiotherapists during lower-limb prosthetic rehabilitation: a thematic analysis. Front Rehabil Sci. (2022) 2:795021. 10.3389/fresc.2021.795021 36188791 PMC9397966

[46] Lefebvre KM Lavery LA. Disparities in amputations in minorities. Clin OrthopRelat Res. (2011) 469(7):1941–50. 10.1007/s11999-011-1842-x PMC3111767 21384209

[47] Miller TA Campbell JH Bloom N Wurdeman SR. Racial disparities in health care with timing to amputation following diabetic foot ulcer. Diabetes Care. (2022) 45(10):2336–41. 10.2337/dc21-2693 36069831 PMC9862414

[48] Raichle KA Hanley MA Molton I Kadel NJ Campbell K Phelps E Prosthesis use in persons with lower- and upper-limb amputation. J Rehabil Res Dev. (2008) 45(7):961–72. 10.1682/jrrd.2007.09.0151 19165686 PMC2743731

[49] Girijala RL Bush RL. Review of socioeconomic disparities in lower extremity amputations: a continuing healthcare problem in the United States. Cureus. (2018) 10(10):e3418. 10.7759/cureus.3418 30542632 PMC6284870

[50] Minc SD Goodney PP Misra R Thibault D Smith GS Marone L. The effect of rurality on the risk of primary amputation is amplified by race. J Vasc Surg. (2020) 72(3):1011–7. 10.1016/j.jvs.2019.10.090 31964567 PMC7404623

[51] Kannenberg A Seidinger S. Health economics: the perspective of a prosthetic manufacturer. J Prosthet Orthot. (2019) 31(1S):49–54. 10.1097/JPO.0000000000000234

[52] Rucker-Whitaker C Feinglass J Pearce WH. Explaining racial variation in lower extremity amputation: a 5-year retrospective claims data and medical record review at an urban teaching hospital. Arch Surg. (2003) 138(12):1347–51. 10.1001/archsurg.138.12.1347 14662537

[53] Eslami MH Zayaruzny M Fitzgerald GA. The adverse effects of race, insurance status, and low income on the rate of amputation in patients presenting with lower extremity ischemia. J Vasc Surg. (2007) 45(1):55–9. 10.1016/j.jvs.2006.09.044 17210382

[54] Edward J Wiggins A Young MH Rayens MK. Significant disparities exist in consumer health insurance literacy: implications for health care reform. Health Lit Res Pract. (2019) 3(4):e250–8. 10.3928/24748307-20190923-01 31768496 PMC6831506

[55] Ortiz SE Segel JE Tran LM. Health savings plans and disparities in access to care by race and ethnicity. Am J Prev Med. (2021) 61(2):E81–92. 10.1016/j.amepre.2021.02.020 33985836

[56] Ingham M Sadik K Zhao X Song J Fendrick AM. Assessment of racial and ethnic inequities in copay card utilization and enrollment in copay adjustment programs. J Manag Care Spec Pharm. (2023) 29(9):1084–92. 10.18553/jmcp.2023.23021 37548953 PMC10510673

[57] Finco MG Sumien N Moudy SC. Clinical evaluation of fall risk in older adults who use lower-limb prostheses: a scoping review. J Am Geriatr Soc. (2023) 71(3):959–67. 10.1111/jgs.18223 36648090 PMC10023358

[58] Finco M Moudy SC Patterson RM. Normalized kinematic walking symmetry data for individuals who use lower-limb prostheses: considerations for clinical practice and future research. J Prosthet Orthot. (2023) 35(1):e1–17. 10.1097/JPO.0000000000000435 37008386 PMC10062529

[59] Finco MG Finnerty C Ngo W Menegaz RA. Indications of musculoskeletal health in deceased male individuals with lower-limb amputations: comparison to non-amputee and diabetic controls. Sci Rep. (2023) 13(1):8838. 10.1038/s41598-023-34773-w 37258530 PMC10232508

[60] Rosen RE Morgan SJ Hafner BJ McDonald CL. Race and ethnicity reporting in contemporary limb loss literature: a scoping review [accepted]. American Academy of Orthotists & Prosthetists 50th Annual Meeting & Scientific Symposium; March 6–9, 2024; Chicago, IL

[61] Andrysek J Christensen J Dupuis A. Factors influencing evidence-based practice in prosthetics and orthotics. Prosthet Orthot Int. (2011) 35(1):30–8. 10.1177/0309364610389353 21515887

[62] Geil MD. Assessing the state of clinically applicable research for evidence-based practice in prosthetics and orthotics. J Rehabil Res Dev. (2009) 46(3):305–13. 10.1682/JRRD.2008.02.0019 19675984

[63] van Twillert S Geertzen J Hemminga T Postema K Lettinga A. Reconsidering evidence-based practice in prosthetic rehabilitation: a shared enterprise. Prosthet Orthot Int. (2013) 37(3):203–11. 10.1177/0309364612459541 23064358

[64] Ramstrand N. Translating research into prosthetic and orthotic practice. Prosthet Orthot Int. (2013) 37(2):108–12. 10.1177/0309364612451268 22868379

[65] Christensen J Andrysek J. Examining the associations among clinician demographics, the factors involved in the implementation of evidence-based practice, and the access of clinicians to sources of information. Prosthet Orthot Int. (2012) 36(1):87–94. 10.1177/0309364611431479 22173642

[66] Bar A Ishai G Meretsky P Koren Y. Adaptive microcomputer control of an artificial knee in level walking. J Biomed Eng. (1983) 5(2):145–50. 10.1016/0141-5425(83)90034-1 6855215

[67] Aeyels B Peeraer L Vander Sloten J Van der Perre G. Development of an above-knee prosthesis equipped with a microcomputer-controlled knee joint: first test results. J Biomed Eng. (1992) 14(3):199–202. 10.1016/0141-5425(92)90052-m 1588776

[68] Berry D. Microprocessor prosthetic knees. Phys Med Rehabil Clin N Am. (2006) 17(1):91–113. 10.1016/j.pmr.2005.10.006 16517347

[69] Centers for Medicare & Medicaid Services. *Medicare Program; Establishment of Special Payment Provisions and Requirements for Qualified Practitioners and Qualified Suppliers of Prosthetics and Custom-Fabricated Orthotics*. (2005). Available at: https://www.cms.gov/Medicare/Provider-Enrollment-and-Certification/MedicareProviderSupEnroll/Downloads/CMS-6012-P_HCPCS_Code_List.pdf (Accessed November 9, 2023). 26110197

[70] Jayaraman C Mummidisetty CK Albert MV Lipschutz R Hoppe-Ludwig S Mathur G Using a microprocessor knee (C-leg) with appropriate foot transitioned individuals with dysvascular transfemoral amputations to higher performance levels: a longitudinal randomized clinical trial. J Neuroeng Rehabil. (2021) 18(1):88. 10.1186/s12984-021-00879-3 34034753 PMC8146219

[71] Biddiss E McKeever P Lindsay S Chau T. Implications of prosthesis funding structures on the use of prostheses: experiences of individuals with upper limb absence. Prosthet Orthot Int. (2011) 35(2):215–24. 10.1177/0309364611401776 21515898

[72] Kannenberg A Seidinger S. Health economics in the field of prosthetics and orthotics: a global perspective. Can Prosthet Orthot J. (2021) 4(2):35298. 10.33137/cpoj.v4i2.35298 37615010 PMC10443514

[73] Fish D. The development of coverage policy for lower extremity prosthetics: the influence of the payer on prosthetic prescription. J Prosthet Orthot. (2006) 18(6):125–9. 10.1097/00008526-200601001-00017

[74] Bekrater-Bodmann R. Factors associated with prosthesis embodiment and its importance for prosthetic satisfaction in lower limb amputees. Front Neurorobot. (2020) 14:604376. 10.3389/fnbot.2020.604376 33519413 PMC7843383

[75] Resnik L Klinger S Gill A Ekerholm Biester S. Feminine identity and functional benefits are key factors in women’s decision making about upper limb prostheses: a case series. Disabil Rehabil Assist Technol. (2019) 14(2):194–208. 10.1080/17483107.2018.1467973 29741966

[76] Moradi-Hades A Farmani F Mardani MA Bahramizadeh M Heidarimoghadam R. The comparative effect of cosmetic and mechanical prosthesis on quality of life and performance in people with medium-length below-elbow amputation. J Prosthet Orthot. (2019) 31(2):89–94. 10.1097/JPO.0000000000000250

[77] Carroll ÁM Fyfe N. A comparison of the effect of the aesthetics of digital cosmetic prostheses on body image and well-being. J Prosthet Orthot. (2004) 16(2):66–8. 10.1097/00008526-200404000-00007

[78] Kaczkowski M. Cosmesis is Much More than Appearance…It’s Function. Washington DC: Amputee Coalition (1999). p. 1–48. Available at: https://www.amputee-coalition.org/wp-content/uploads/2015/03/cosmesis1.pdf (Accessed November 09, 2023).

[79] Mayo AL Gould S Cimino SR Glasford S Harvey E Ratto M A qualitative study on stakeholder perceptions of digital prosthetic socket fabrication for transtibial amputations. Prosthet Orthot Int. (2022) 46(6):607–13. 10.1097/PXR.0000000000000157 36515905

[80] International Organization for Standardization. *Prosthetics and Orthotics (ISO/TC 168)*. (1977). Available at: https://www.iso.org/committee/53630/x/catalogue/ (Accessed November 9, 2023).

[81] U.S. Food and Drug Administration. *Physical Medicine Prosthetic Devices (21CFR890)*. (2023). Available at: https://www.accessdata.fda.gov/scripts/cdrh/cfdocs/cfcfr/CFRSearch.cfm?CFRPart=890&showFR=1&subpartNode=21:8.0.1.1.32.4 (Accessed November 9, 2023).

[82] Heim S. Advances in prosthetic and orthotic education and training in developing countries: a personal view. Prosthet Orthot Int. (1995) 19(1):20–30. 10.3109/03093649509078228 7617455

[83] Marino M Pattni S Greenberg M Miller A Hocker E Ritter S Access to prosthetic devices in developing countries: pathways and challenges. In: 2015 IEEE Global Humanitarian Technology Conference (GHTC). (2015). p. 45–51. 10.1109/GHTC.2015.7343953

[84] Spaulding SE Kheng S Kapp S Harte C. Education in prosthetic and orthotic training: looking back 50 years and moving forward. Prosthet Orthot Int. (2020) 44(6):416–26. 10.1177/0309364620968644 33164659

[85] World Health Organization. *WHO Standards for Prosthetics and Orthotics*. (2017). Available at: https://www.who.int/publications/i/item/9789241512480 (Accessed November 9, 2023).

[86] International Society for Prosthetics and Orthotics. *ISPO Education Standards for Prosthetic Orthotic Occupations*. (2018). Available at: https://www.ispoint.org/activities/education-accreditation/education-standards/ (Accessed November 9, 2023).

[87] National Commission on Orthotic and Prosthetic Education. *CAAHEP Standards*. (2018). Available at: https://ncope.org/index.php/home-page-v2/academic-programs/institution-educator-info/caahep-standards-request-for-accreditation-services/ (Accessed November 9, 2023).

[88] Cochrane H Rusaw D Mullen A Spaulding S Brinkman J. Evidence-based practice in education for prosthetic orthotic occupations. ISPO 19th World congress; April 24–27, 2023; Guadalajara, Mexico. 10.1097/PXR.0000000000000240

[89] Wright D Mullen A Gardner A. Does student-led faculty development have A place in health professions education? [version 1]. MedEdPublish. (2019) 8(34):1–14. 10.15694/mep.2019.000034.1 38089393 PMC10712635

[90] Cruz MLC Utay JB Mullen AH. Entrustment trends in orthotic and prosthetic residencies. Prosthet Orthot Int. (2020) 44(2):73–80. 10.1177/0309364620909236 32133918

[91] Anderson CB Fatone S Mañago MM Swink LA Hager ER Kittelson AJ Improving shared decision-making for prosthetic care: a qualitative needs assessment of prosthetists and new lower-limb prosthesis users. Prosthet Orthot Int. (2023) 47(1):26–42. 10.1097/PXR.0000000000000142 35622457 PMC9691789

[92] Quigley M Dillon MP Fatone S. Development of shared decision-making resources to help inform difficult healthcare decisions: an example focused on dysvascular partial foot and transtibial amputations. Prosthet Orthot Int. (2018) 42(4):378–86. 10.1177/0309364617752984 29393805

[93] Anderson CB Kittelson AJ Wurdeman SR Miller MJ Stoneback JW Christiansen CL Understanding decision-making in prosthetic rehabilitation by prosthetists and people with lower limb amputation: a qualitative study. Disabil Rehabil. (2023) 45(4):723–32. 10.1080/09638288.2022.2037745 35389313 PMC9537359

[94] Dillon MP Quigley M Fatone S. Outcomes of dysvascular partial foot amputation and how these compare to transtibial amputation: a systematic review for the development of shared decision-making resources. Syst Rev. (2017) 6(1):54. 10.1186/s13643-017-0433-7 28288686 PMC5348872

[95] Straus SE Tetroe J Graham I. Defining knowledge translation. CMAJ. (2009) 181(3–4):165–8. 10.1503/cmaj.081229 19620273 PMC2717660

[96] Graham ID Harrison MB, Logan J the KT Theories Research Group. A review of planned change (knowledge translation) models, frameworks and theories. JBI International Convention; November 28–30, 2005; Adelaide, Australia.

[97] Finco M Patterson RM Moudy SC. A pilot case series for concurrent validation of inertial measurement units to motion capture in individuals who use unilateral lower-limb prostheses. J Rehabil Assist Technol Eng. (2023) 10:20556683231182322. 10.1177/20556683231182322 37441370 PMC10334000

[98] Miller KT Russell M Jenks T Surratt K Poretti K Eigenbrot SS The feasibility and validity of a wearable sensor system to assess the stability of high-functioning lower-limb prosthesis users. J Prosthet Orthot. (2020) 33(3):213–22. 10.1097/JPO.0000000000000332 PMC7839069 33510564

[99] Rattanakoch J Samala M Limroongreungrat W Guerra G Tharawadeepimuk K Nanbancha A Validity and reliability of inertial measurement unit (IMU)-derived 3D joint kinematics in persons wearing transtibial prosthesis. Sensors (Basel). (2023) 23(3):1738. 10.3390/s23031738 36772783 PMC9920655

[100] Choo YJ Chang MC. Use of machine learning in the field of prosthetics and orthotics: a systematic narrative review. Prosthet Orthot Int. (2023) 47(3):226–40. 10.1097/PXR.0000000000000199 36811961

[101] Reddy S Allan S Coghlan S Cooper P. A governance model for the application of AI in health care. J Am Med Inform Assoc. (2020) 27(3):491–7. 10.1093/jamia/ocz192 31682262 PMC7647243

[102] Esmaeilzadeh P Mirzaei T Dharanikota S. Patients’ perceptions toward human-artificial intelligence interaction in health care: experimental study. J Med Internet Res. (2021) 23(11):e25856. 10.2196/25856 34842535 PMC8663518

[103] Tulk Jesso S Kelliher A Sanghavi H Martin T Henrickson Parker S. Inclusion of clinicians in the development and evaluation of clinical artificial intelligence tools: a systematic literature review. Front Psychol. (2022) 13:830345. 10.3389/fpsyg.2022.830345 35465567 PMC9022040

[104] Finco MG Mir N Gresham G Huisingh-Scheetz M. Ethical considerations for digital health technology in older adult care. Lancet Healthy Longev. (2024) 5(1):12–3. 10.1016/S2666-7568(23)00236-2 PMC11185426 38183992

[105] de Hond AAH van Buchem MM Hernandez-Boussard T. Picture a data scientist: a call to action for increasing diversity, equity, and inclusion in the age of AI. J Am Med Inform Assoc. (2022) 29(12):2178–81. 10.1093/jamia/ocac156 36048021 PMC9667164

[106] Isaacs-Itua A Sedki I. Management of lower limb amputations. Br J Hosp Med. (2018) 79(4):205–10. 10.12968/hmed.2018.79.4.205 29620980

[107] Keszler MS Wright KS Miranda A Hopkins MS. Multidisciplinary amputation team management of individuals with limb loss. Curr Phys Med Rehabil Rep. (2020) 8(3):118–26. 10.1007/s40141-020-00282-4

